# Deep Assessment of Genomic Diversity in Cassava for Herbicide Tolerance and Starch Biosynthesis

**DOI:** 10.1016/j.csbj.2017.01.002

**Published:** 2017-01-14

**Authors:** Jorge Duitama, Lina Kafuri, Daniel Tello, Ana María Leiva, Bernhard Hofinger, Sneha Datta, Zaida Lentini, Ericson Aranzales, Bradley Till, Hernán Ceballos

**Affiliations:** a Agrobiodiversity Research Area, International Center for Tropical Agriculture (CIAT), Cali, Colombia; b Plant Breeding and Genetics Laboratory, Joint FAO/IAEA Division, International Atomic Energy Agency, Seibersdorf, Austria; c Department of Biological Sciences, School of Natural Sciences, Universidad Icesi, Cali, Colombia; d Systems and Computing Engineering Department, Universidad de los Andes, Bogotá, Colombia

**Keywords:** Cassava, Pooled targeted resequencing, Herbicide tolerance, Starch biosynthesis, SNP detection

## Abstract

Cassava is one of the most important food security crops in tropical countries, and a competitive resource for the starch, food, feed and ethanol industries. However, genomics research in this crop is much less developed compared to other economically important crops such as rice or maize. The International Center for Tropical Agriculture (CIAT) maintains the largest cassava germplasm collection in the world. Unfortunately, the genetic potential of this diversity for breeding programs remains underexploited due to the difficulties in phenotypic screening and lack of deep genomic information about the different accessions. A chromosome-level assembly of the cassava reference genome was released this year and only a handful of studies have been made, mainly to find quantitative trait loci (QTL) on breeding populations with limited variability. This work presents the results of pooled targeted resequencing of more than 1500 cassava accessions from the CIAT germplasm collection to obtain a dataset of more than 2000 variants within genes related to starch functional properties and herbicide tolerance. Results of twelve bioinformatic pipelines for variant detection in pooled samples were compared to ensure the quality of the variant calling process. Predictions of functional impact were performed using two separate methods to prioritize interesting variation for genotyping and cultivar selection. Targeted resequencing, either by pooled samples or by similar approaches such as Ecotilling or capture, emerges as a cost effective alternative to whole genome sequencing to identify interesting alleles of genes related to relevant traits within large germplasm collections.

## 1 Introduction

Cassava is one of the most important crops in the tropics, surpassed only by maize and rice [1], and it is usually grown by poor farmers living in marginal and submarginal lands of the tropics [2]. It provides staple food for over 700 million people in Africa (51%), Asia (29%) and South America (20%) [3], being their main source of carbohydrates, in part due to its capacity to produce more energy per hectare than other crops [4], [5]. Cassava is also preferred among other crops in these areas because it keeps competitive yields under poor soils, drought, acidic conditions, high air temperatures and evapotranspiration, pests, and diseases [6], [7], [8]. In marginal areas where grain crops often fail, cassava can strive, allowing farmers to harvest it when needed [9], [10].

**Fig. 1:**
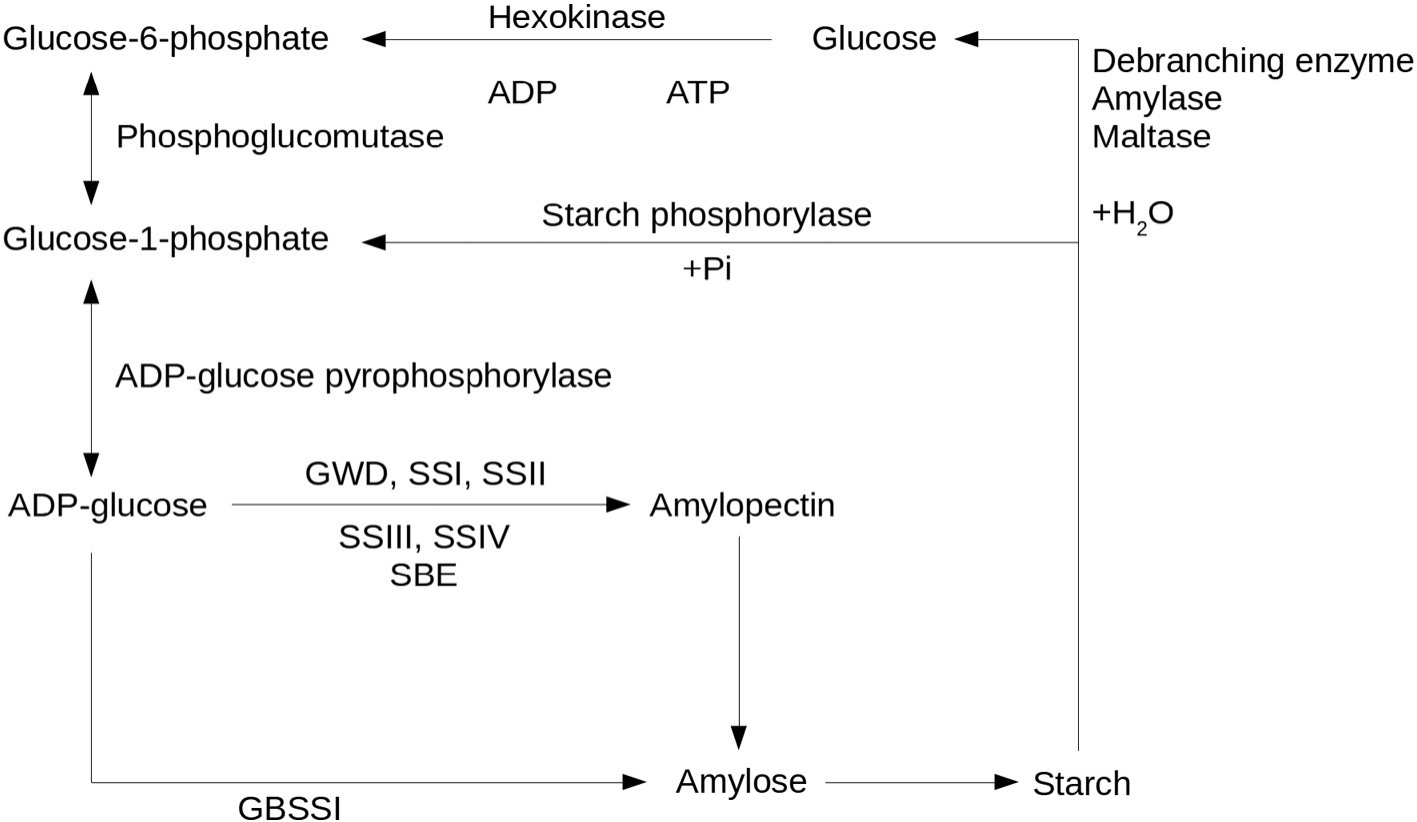
Metabolic reactions related to starch biosynthesis. Arrows indicate reactions catalyzed by the enzymes listed close to the corresponding arrow.

In addition to human and animal consumption, cassava has great potential as a source of industrial starch [11]. In fact, cassava is the second most important source of starch worldwide. In the last two decades, cassava production has increased mainly owing to its superior starch quality; which is used primarily in food-processing, paper, glue, textiles, and pharmaceutical industries or occasionally for ethanol production [8]. Therefore one important goal of cassava breeding programs is to develop new varieties with high starch content [12] and with variation in its starch functional properties [13], [14]. The biosynthesis of starch involves the production of amylose and amylopectin molecules, which is catalyzed by a series of enzymes (Fig. 1). The synthesis of amylose is catalyzed by the *GBSSI* (Granule bound starch synthase) enzyme [15]. Mutations that knock out this protein are known as *waxy* mutations, because the resulting starches lack amylose [16]. There is a whole complex of enzymes involved in the synthesis of amylopectin: four soluble starch synthases (*SSI*, *SSII*, *SSIII* and *SSIV*), two types of starch branching enzymes (*SBEI* and *SBEII*), the Glucan Water Dikinase (*GWD*), and various debranching enzymes and kinases [17]. The SS and the SBE enzymes contribute glucose units to the main chain, and mediate the cleavage and branch formation of the amylopectin units [18]. Alteration in SBE activity affects the number of and size distribution of amylopectin branches [17]. It is hard to determine the exact role of each isoform of the soluble starch synthases in this process due to their different gene expression, which depends on both genotypic and environmental variations [18]. GWD controls the overall rate of starch breakdown with a central rate limiting role in starch breakdown machinery and downstream starch synthesis [19]. Plants lacking this protein accumulate abnormally high levels of starch [20].

Another central goal in cassava breeding is the development of herbicide-tolerant cultivars, because the use of herbicides is an effective mechanism to control weeds, reducing labor and alleviating problems of soil erosion associated with mechanical weeding [21]. Studies on the impact of introducing herbicide resistance cassava in Colombia estimated production cost savings between 15% and 25% [22]. Additionally, the positive environmental effects which reduce tillage would bring for increased sustainability of the crop on marginal lands [23].

Resistance to two types of herbicides, inhibiting amino acid biosynthesis, has been commercially exploited in different crops and was targeted in this study. The first group of herbicides (imidazolinones, sulfonylureas, triazolopyrimidine, pyrimidinyl-thiobenzoates, and sulphonyl-aminocarbonyl-triazolinone), interact with the enzymes Acetohydroxyacid synthase (AHAS) and acetolactate synthase (ALS) [24], [25]. AHAS has an important role during the synthesis of branched chain amino acids such as valine, leucine, and isoleucine, which are important for the synthesis of several proteins [24]. However, variations in just one amino acid in the binding site of AHAS enzymes can lead to a change in their quaternary structure, blocking herbicide binding and conferring tolerance in the plant. At least five naturally occurring mutations in AHAS, leading to resistance, have been reported in different plant species [24]. The second class of herbicides also affecting amino acid synthesis is the PPT (l-phosphinothricin), also known as glufosinate, and act on the glutamine synthase enzyme (GS). GS synthesizes glutamine and is very important in the regulation of the nitrogen metabolism [26], [27]. With the development of transgenic technology, studies established a protocol of using somatic cotyledons as explants for the transformation of cassava [28] successfully transformed a herbicide-resistance gene into the cotyledons of cassava Per 183 by the Agrobacterium mediated method [21]. However, the development of transgenic herbicide-resistant cassava faces regulatory problems that have restricted the adoption of the technology in Africa (with the exception of South Africa).

CIAT holds in trust the largest global germplasm collection of cassava and other *Manihot* species (more than 6000 accessions). The *in vitro* collection at CIAT was initiated in 1978 soon after the technology for slow growth *in vitro* became available [29]. The germplasm collection is a valuable asset and the main repository of genetic variability of cassava. Advanced materials developed from it were the sources of amylose free starch mutations [14]. Although these discoveries provided important proof of the value of the collection, it also highlighted the limited exploration and exploitation of its genetic variability. This work also highlighted how time consuming and inefficient it is to expose useful recessive traits by conventional self-pollination methods. A recent partial screening of the collection allowed discovering varieties carrying two mutations responsible for improved starch quality traits [30]. These findings are encouraging to explore cost-effective alternatives to screen the germplasm collection in search for useful mutations for agronomically relevant traits.

In recent years, the development of high throughput sequencing technologies led to major progress in the understanding of genomic variation in plants, increasing the number of sequenced genomes [31]. However, despite the economic importance of cassava, studies of its genomic diversity are much less complete, compared to other crops such as rice, wheat or maize. Up-to-date the largest study of genomic variability in cassava, which includes 1280 accessions, is based on 402 single nucleotide polymorphisms (SNPs) scattered across the genome [32]. Although a draft cassava genome was assembled and made available in 2012 [33], a chromosome-level assembly was only achieved in 2016 [34]. In the meantime, genotyping by sequencing (GBS) has been a commonly used alternative to obtain dense datasets of genome-wide SNP markers [35]. These SNPs have been used to develop saturated genetic maps for breeding populations, genetic mapping of traits [36], [37], [38], and markers for fingerprinting [39]. More recently they have been used to perform a Genome-wide Association Study (GWAS) to identify loci related to resistance to the Cassava mosaic disease [40]. Although GBS is an efficient technique to screen markers and gather information across the genome, it does not allow the study and discovery of variability within specific genes. Sequencing of RNA has also been used as an alternative to identify expressed variation across thousands of genes [41]. However, the cost per sample of this technique is still prohibitive for large numbers of samples. For this reason, targeted resequencing remains an alternative approach to study genetic variability in specific loci.

In this study, we performed pooled targeted resequencing of DNA from 1667 cassava accessions to detect rare SNPs in specific genes associated with the starch biosynthesis pathway and with herbicide resistance. Selected accessions represent about one fourth of the entire collection and include landraces from the most important regions of cassava production in Latin America. We combined the results of 7 variant calling tools applied to aligned reads obtained with two different algorithms to develop a dataset of more than 2000 SNPs within the genes of interest. These SNPs can be prioritized and validated for allele mining and efficient identification of mutated genes in accessions within the cassava germplasm collection.

## 2 Results

### 2.1 Targeted Pooled Sequencing of the Cassava Germplasm Bank

DNA was extracted from a total of 1728 accessions from the germplasm collection (Supplementary Table 1). In general, DNA quality was good, with 70% of the samples showing clear shaped bands without significant smearing (Supplementary figure 1). Only 61 samples were discarded due to low DNA concentration. For pooled resequencing two possible methods to normalize DNA concentration across samples were evaluated: the use of paramagnetic beads and visual concentration determination using agarose gels (see Section 4.2). Owing to inconsistencies observed when using beads, a 96 well agarose gel system was adopted. Based on a literature review and on blast searches of the cassava reference genome [34], a total of 6 genes related to herbicide tolerance and 8 genes related to starch biosynthesis were chosen for this study (Supplementary Table 2). To capture the exonic regions of the targeted genes, a total of 121 primer pairs having an expected amplicon length of 600 bp, were designed (Supplementary Table 3). This resulted in an expected total length of 72 kbp of DNA sequence targeted in the assay. To assess the quality of these primers, PCR assays were performed on one of the pooled samples. Only 18 primers failed to amplify, 13 of them located within the gene GWD (Supplementary figure 2).

**Fig. 2:**
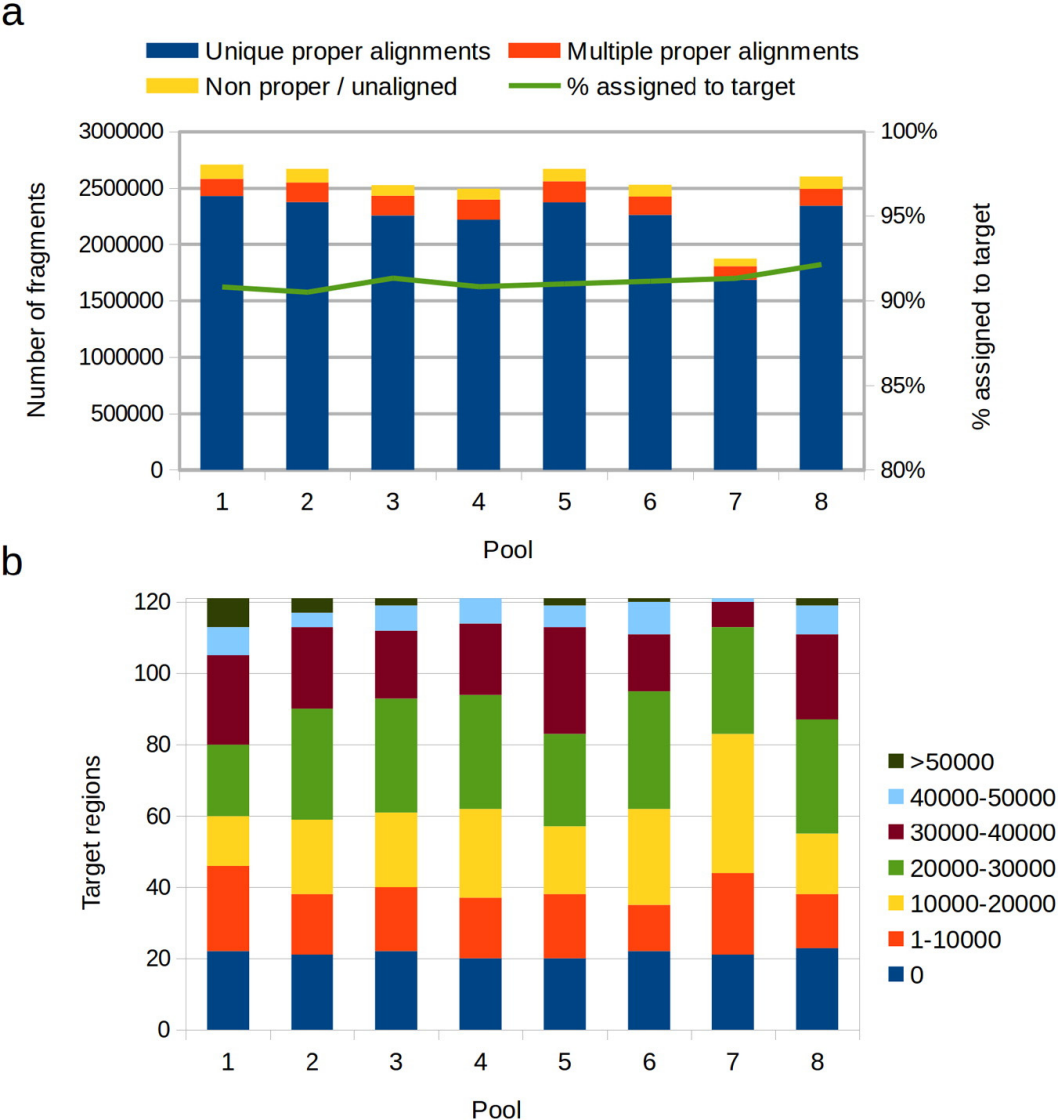
Read alignment statistics per pool. a) Number of fragments sequenced as paired-end reads for each pool. Counts are discriminated as number of fragments aligning with the expected distance and orientation (proper pair) to a unique region of the genome, fragments aligning as a proper pair to multiple regions and fragments not aligned or not aligned as a proper pair. The line indicates the percentage of fragments that could be uniquely assigned to a targeted region defined by the coordinates of its corresponding primer pair. b) Distribution of the number of fragments assigned to each target region within each pool.

Amplicon products for each pool were sent to the high throughput sequencing Illumina MiSeq instrument available at the Plant Breeding and Genetics Laboratory from the International Atomic Energy Agency (IAEA) in Seibersdorf, Austria. After one 2 × 300 paired-end sequencing run, around 2.5 million fragments were obtained for each pool. Assuming that these fragments are evenly distributed across the targeted regions, this raw sequencing production represents a expected read depth of around 20,000 × per targeted base pair within each pool. Reads were trimmed to 240 bp for the first read and to 170 bp for the second read to remove low quality ends. Alignment of the trimmed reads to the reference genome yielded an overall alignment rate of 97%, with 89% of the fragments aligning to unique locations and with the expected distance and orientation (Fig. 2a). Even requiring a stringent reciprocal overlapping of 90% between each aligned fragment and a targeted region, 91% of the total fragments could be reliably assigned to a single region defined by one primer pair (Supplementary Table 3). This percentage represents the capture success rate of the experiment. Moreover, fragments within each pool were assigned more or less evenly to the targeted regions for which primer amplification was successful (Fig. 2b). Besides 17 of the 18 primers for which amplification failed, only five additional primers had less than 20 reads assigned within each pool. Except for the case of pool 7, more than half of the regions had more than 20,000 fragments assigned within each pool. Pool 7 had only 38 regions with this minimum read depth because about 600,000 fewer fragments were sequenced for this pool. In principle, each fragment assigned to a region represents one read of the entire region. However, the initial trimming performed on each read reduced the sequenced portion of its corresponding region, leaving uncovered the central parts of some of the regions (Supplementary figure 4).

### 2.2 Comparison of Tools for SNP Discovery in Pooled Data

The number of fragments assigned to each region is tightly related to the total read depth available within each particular locus to assess the presence of non-reference alleles, call variation, and estimate relative allele frequencies based the number of reads supporting each allele. Theoretically, if 10,000 fragments are assigned to one region within one pool, the minor allele of a biallelic variant with a frequency of 0.01 within the samples included in the pool should be observed in about 100 reads. Because about 200 samples were included in each pool, heterozygous variants present in only one sample would have a minor allele frequency (MAF) of 1/400 = 0.0025 within one pool. Although in this experiment some of these variants would have enough read support be detected, it becomes increasingly difficult to separate the support of true alleles with low frequency from sequencing errors.

**Fig. 3:**
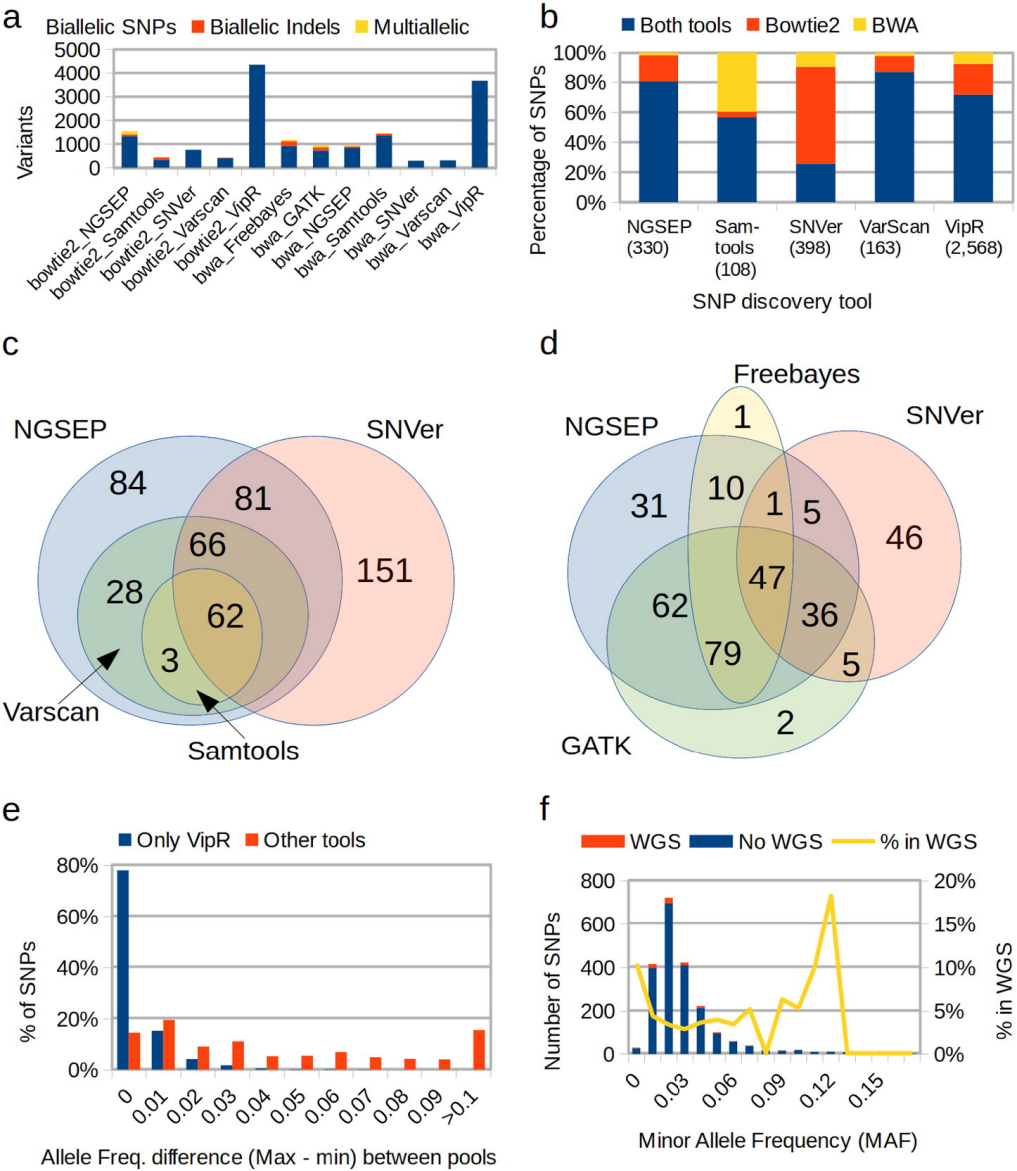
Comparison of variant calls with different pipelines. a) Number of total variants detected by each variant caller; b) Comparison of number of SNPs called by each SNP discovery tool on alignments obtained with bowtie2 and with BWA; c) Comparison of number of SNPs called between different SNP calling tools on bowtie2 alignments; d) Comparison of number of SNPs called between different SNP calling tools on BWA alignments; e) Distribution of differences in predicted alternative allele frequency between pools for the curated dataset of SNPs; f) Distribution of minor allele frequency for SNPs identified only by VipR discriminating SNPs found in a dataset of variants obtained from WGS data. The line indicates the percentage of such SNPs within each category.

To identify sites with evidence of variation within the pools, we combined the results of 12 previously published bioinformatic pipelines designed to discover single nucleotide polymorphisms (SNPs) and in some cases small indels. The pipelines are the combination of 2 read alignment tools, Bowtie2 [42] and the Burrows-Wheeler Aligner (BWA) [43] with 7 variant discovery programs: Freebayes [44], the Genome Analysis Toolkit (GATK) [45], the Next Generation Sequencing Experience Platform (NGSEP) [46], Samtools [47], SNVer [48], VarScan [49] and VipR [50]. From these tools, SNVer and VipR were particularly designed to identify variation in pools. Because Freebayes and GATK presented problems or were not compatible with bowtie2 alignments, we only ran these tools using as input BWA alignments. On average 1350 variants (1270 SNPs) were predicted within each pool, being SNVer on BWA alignments the pipeline reporting the smallest number of SNPs (294) and VipR on bowtie2 alignments the pipeline reporting the largest number (4354) (Fig. 3a). The average number of indels was 80. VipR and SNVer were not able to detect any indel and VarScan detected indels only from bowtie2 alignments.

Merging the variants predicted by the different pipelines, a raw dataset of 7925 variants was obtained, including 7348 biallelic SNPs, 258 biallelic indels and 319 multiallelic variants. Reads supporting each allele of each variant within each pool were counted following the genotyping step of the NGSEP pipeline and allele frequencies were estimated from these counts. About 70% of the raw variants are located within the targeted regions. At first sight, this percentage looks inconsistent with the capture success rate of 91% reported above. The explanation for this outcome is that variants outside targeted regions are called from the few reads falling away from targeted regions and then the total read depth of those variants is much lower than that of the variants within the targeted regions (Supplementary figure 3). The raw variants were filtered by minimum read depth, number of pools in which the variant is observed, and minimum alternative allele frequency. To differentiate true rare SNPs from sequencing errors, the number of errors for each raw SNP was estimated as the average between the third and the fourth smallest allele read depth. Then, the ratio between the read depth of the allele with the second count and the estimated number of sequencing errors was calculated and the SNP was filtered out if this ratio was less than 5. This filtering procedure yielded a curated dataset of 2614 SNPs (Supplementary Table 4). Estimated allele frequencies for curated SNPs were adjusted taking into account read counts of the two predicted alleles. Contrasting the raw calls obtained using each tool during the discovery step with this filtered dataset, we found that 80% of the SNPs in the final set were discovered only by VipR and only 46 SNPs were reported by tools different than VipR. The filters reduced the number of SNPs called by each method to about half in the case of vipR and SNVer, and up to 1 over 10 in the case of Samtools. Samtools only reported 108 of the filtered SNPs with only one SNP not shared by other tools. SNVer and NGSEP were the second and third tools reporting more SNPs within this dataset with 398 and 330 SNPs respectively. The SNPs contributed by the same discovery tool using different read alignment methods were compared to assess the consistency of each method relative to the input alignments (Fig. 3b). Although Varscan only called a total of 163 SNPs, 87% of them were consistently called from bowtie2 and BWA alignments. 80% of the SNPs called by NGSEP were consistent across alignment tools. The smallest percentage of intersection (25.6%) was reported by SNVer. With the exception of Samtools, the other tools reported more SNPs using bowtie2 alignments than BWA alignments.

In absence of a gold-standard to perform a formal quality assessment of the variants predicted by different pipelines, we also calculated the intersections between SNP discovery tools, excluding vipR (Fig. 3c and d). Starting from alignments built using bowtie2, Varscan calls every SNP called by Samtools, and NGSEP calls every SNP called by Varscan or by Samtools. NGSEP and SNVer share 209 SNPs, which represents the 58% of the SNPs called by SNVer and the 64% of the SNPs called by NGSEP. Starting from BWA alignments the sharing between the same 4 tools remains consistent, with the exception of one SNP called by Samtools, which is not called by any other tool (including vipR) and four SNPs called by samtools, NGSEP and SNVer and not called by Varscan. Every SNP called by Varscan is also called by NGSEP. GATK and Freebayes were added to the comparison performed starting from BWA alignments. 47 SNPs were identified by the four methods and 117 additional SNPs were called by three out of four methods. The number of shared SNPs between NGSEP and SNVer (89) still represents 63% of the total SNPs called by SNVer. However, in this case the same number only represents 33% of the SNPs called by NGSEP. From the 182 SNPs called by NGSEP and not called by SNVer, 83% are called either by GATK or by Freebayes.

We also investigated the consistency of allele frequency estimations between pools, taking into account that the samples were pooled without information of population structure and hence the allele frequencies of variants should be stable across pools. Fig. 3e shows that the differences between the largest and the smallest predicted allele frequency for each variant are generally small, having only 213 cases of differences larger than 0.05 and 78 cases of differences larger than 0.1. Because the set of SNPs identified in this study is largely dominated by the SNPs only identified by VipR, this comparison was performed independently for the SNPs predicted only by VipR and for the SNPs predicted by at least one of the other tools. As expected, the subset of variants only called by vipR consists on SNPs with low MAF (Fig. 3f). Overall, this result indicates that the predictions are stable, especially for the SNPs with high MAF in which large errors on the prediction of allele frequencies could be expected. The largest difference was observed in the SNP located at 27,238,423 of chromosome 3. Whereas the alternative allele (Guanine) is predominant in pool 4 with 27,876 reads supporting this allele and only 989 reads supporting the alternative allele (Adenine), in pool 8 the alternative allele is supported by only 6 reads, which is much smaller than the read support of the reference allele (13,028) and it is even smaller than the read counts for cytosine and thymine (9 and 13 respectively). Read counts in the other pools are relatively balanced between the reference and the alternative allele.

Looking for further evidence to assess the precision of the SNP calling procedure, we compared the SNPs predicted in this work with the SNPs identified from an analysis of whole genome sequencing (WGS) data from 58 cassava varieties [34]. Due to the reduced number of samples, it would be expected that most SNPs with low MAF would not be observed in the WGS panel. However, to the best of our knowledge, this is the only publicly available dataset of SNPs aligned to the current cassava reference genome. A total of 350 SNPs (13.4%) appear in the two datasets (Supplementary Table 4). Whereas 54.3% (272) of the variants called by at least one of the other tools appear in the WGS dataset, only 3% (78) of the variants predicted only by vipR appear in the WGS dataset. However, these 78 SNPs are not skewed toward the highest MAF ranges within the subset of VipR SNPs, as it would be the case if the SNPs in the lower MAF ranges would be mostly false positives. The SNPs present in the WGS dataset are well distributed across the different ranges of MAF and in particular 10% of the SNPs with MAF less than 0.01 appear in the WGS dataset.

### 2.3 Functional Characterization of Variants within Targeted Genes

**Fig. 4:**
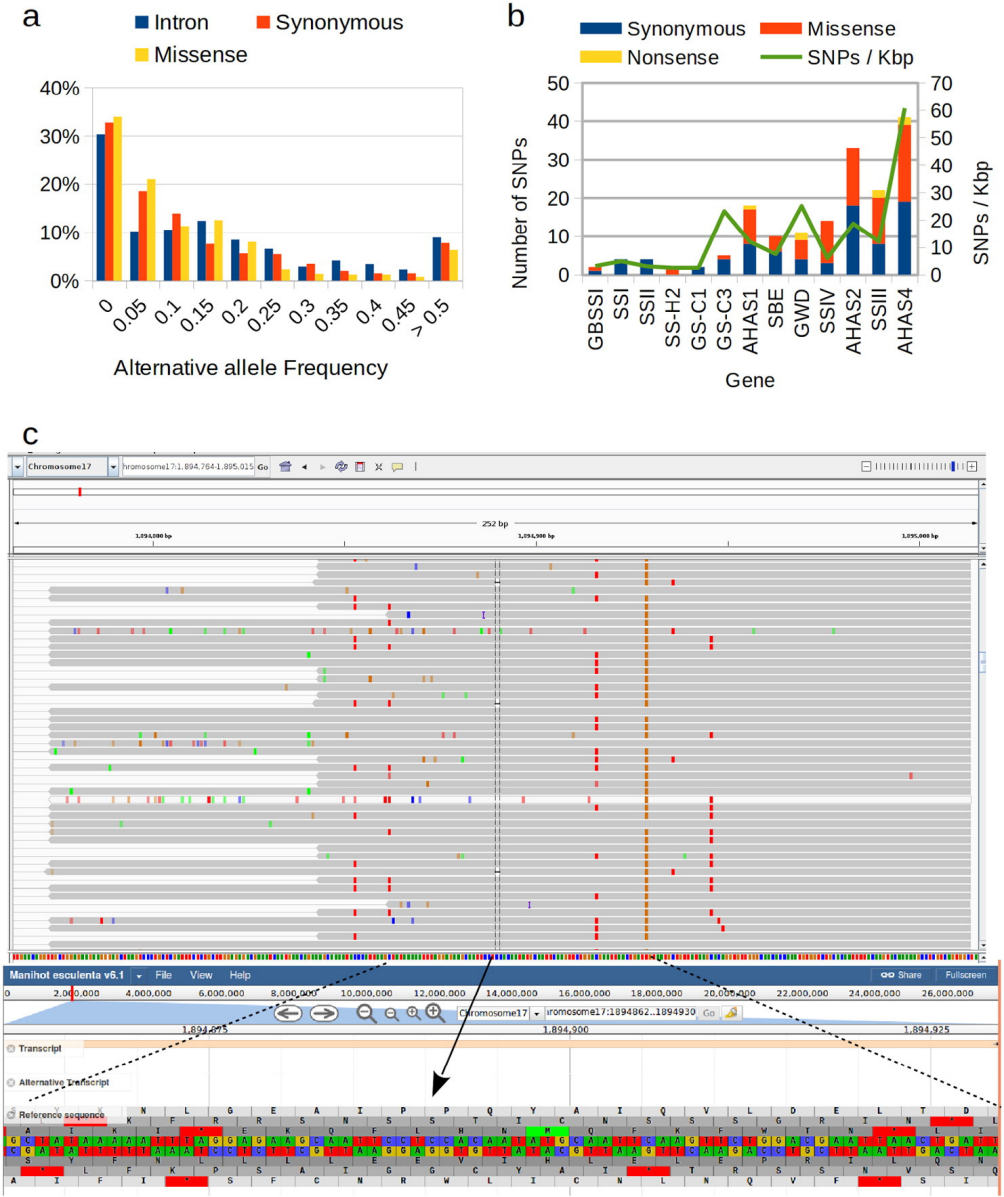
Functional analysis of variants. a) Distribution of alternative allele frequencies observed over the 8 pools for the dataset obtained removing SNPs that were called only by vipR. b) Distribution of SNPs within coding regions of the genes sequenced in this study. The line represents the number of SNPs per kilo base pair c) Reads supporting a 1 bp deletion changing the open reading frame to generate an early stop codon in the allele of the AHAS gene at chromosome 17. The upper panel is a visualization using the integrative genomics viewer (IGV) of the reads spanning the region (gray rectangles). Colors different than gray indicate base calls different than the reference allele. The highlighted column shows reads reporting a 1 bp deletion. The lower panel shows a view of the JBrowse visualizer available in phytozome of the highlighted subregion, including the nucleotide sequence and the six possible amino acid translations. The arrow indicates the location of the frameshift deletion.

Functional annotations of the dataset of filtered SNPs using both NGSEP and SNPeff were performed, obtaining 317 synonymous, 1037 missense and 59 non sense mutations (Fig. 4a). At first sight, the number of missense mutations looks unexpectedly high. However, this can be explained by the accumulation of rare mutations over the varieties sequenced in the pools. Keeping only variants called by at least one method different than VipR, the number of missense mutations (91) becomes similar to the number of synonymous mutations (84). Fig. 4a shows that the percentage of rare variants reduces to 35% and that synonymous mutations and mutations in introns tend to have larger allele frequencies than non-synonymous mutations. Fig. 4b shows the distribution of mutations in coding regions per gene. The AHAS genes accumulate 55% of the mutations and seem to have larger SNP density than the genes related to amylose content, even after normalizing by the length of the covered exonic regions. Within the SS family, *SSIII* and *SSIV* show a larger SNP density and for *SSIV* in particular the number of synonymous mutations (3) is much smaller than the number of missense mutations (11). Six of these missense mutations have a predicted MAF larger than 0.1. The number of non-sense mutations reduced to only seven. Interestingly, two of these mutations, which modify the codons 141 and 143 at exon 4 of the gene GWD showed alternative allele frequencies close to 0.5 and to 0.25 respectively over the 8 pools. Read counts indicate that in almost all pools the alternative alleles of both mutations were supported by over 3000 reads and that the number was always 5-fold higher than the number of reads supporting other alternative allele. Three additional mutations with MAFs larger than 0.15 are located close to the end of the *SSIII* and the *AHAS4* genes.

Unfortunately vipR and SNVer, which were the two software packages implementing models for pooled sequencing data, were not designed to call small indels. Combining results of the other tools, 4 small indels were identified within coding regions of the sequenced genes (Supplementary Table 5). One of these indels, located within the gene SBE was a missense 3 bp deletion, which removes a lysine amino acid. The three remaining indel mutations are all 1 bp deletions located at the AHAS 4 gene located at chromosome 17 (Fig. 4c). The three mutations are predicted to change the open reading frame of the gene, which is likely to produce an early stop codon. Predicted allele frequencies based on read counts indicate that these mutations are present in about 15% of the sequenced cultivars.

## 3 Discussion

The recent releases of chromosome-level assemblies for different plants and the continuous reduction in sequencing costs allows research in staple crops such as cassava to enter the post-genomic era in which comprehensive characterization of genomic diversity across complete genebank collections becomes a feasible task [51]. However, because whole genome sequencing (WGS) costs are still in the order of $500 per sample for cassava, cost-effective sequencing alternatives are preferred for different applications. Genotype by Sequencing (GBS), which recently became the method of choice for applications such as construction of genetic maps, population structure and association mapping, has as main disadvantage that it does not allow to obtain complete sequencing of any single gene. Because the objective in this work was to perform allele mining over the CIAT germplasm collection for genes already known to be related to starch content and herbicide tolerance, we decided to implement a targeted sequencing approach based on PCR assays guided by carefully selected primers. This strategy allowed maximizing the power of high throughput sequencing (HTS) to obtain accurate information of variability across more than 1500 varieties from the germplasm collection. To the best of our knowledge, this study is up-to-date the sequencing effort involving the largest number of samples in cassava.

The targeted sequencing strategy followed in this experiment indeed revealed a large amount of variants at different allele frequencies within the targeted genes. A comparison with the SNPs identified by whole genome sequencing of 58 African varieties (Bredeson, 2016) served as validation of the variants with high Minor Allele Frequency (MAF) but also showed that sequencing a limited number of varieties does not allow identification of a large amount of genetic variation that could be potentially relevant for breeding purposes. The consistency in predictions of allele frequencies observed across the eight pools suggests that the method employed for DNA normalization and the bioinformatic analysis were generally effective and hence they can be used for future pooled sequencing experiments. The main drawback that we could observe using the pooled targeted sequencing approach was a reduction of the regions effectively sequenced by the experiment due to the increased error rates toward the 3′ ends of the reads. Because reads are directly sequenced from PCR products and not randomly sampled within the targeted regions, high error rates at the 3′ end of the reads will accumulate at the central parts of the targeted regions, producing a large amount of false positives. If reads are trimmed to prevent this effect, central parts of some of the targeted regions are lost. In future experiments, amplicon lengths of PCR products should be reduced to take into account the error rate of the sequencing instrument. A second drawback of this approach is that individual genotyping of the variants revealed by the experiment can not be achieved within the experiment. We are currently evaluating different techniques to perform direct genotyping of the most promising SNPs identified in this work.

The most commonly used tools for variants discovery (NGSEP, GATK, Samtools, Freebayes and Varscan) are not designed to detect low frequency variants in pooled samples, because they were designed to perform variants discovery from alignments of reads sequenced from individual samples. Hence, one of the assumptions to improve the genotyping quality in these tools is that the two alleles in heterozygous sites will have even representation in the sample. This is not the normal case for pooled samples because population allele frequencies determine the relative proportion of read counts supporting each allele within variant sites. However, we could only find two additional software tools (VipR and SNVer) that would be feasible to run on current aligned HTS reads and that implemented statistical models to find the low frequency variants that could potentially be extracted from these data. An initial comparison of the variants obtained with these two tools showed that their results were very divergent, with VipR reporting between five and twelve times more variants than SNVer, depending on the read alignment tool (Fig. 3a). Although SNVer could effectively identify some low frequency variants that the other pipelines could not identify, these variants were not consistently identified across read alignment tools. Moreover, SNVer missed some variants with large frequency that could be discovered even with the traditional tools. On the other hand, manual examination of the read counts for some of the raw SNPs with low frequency alternative nucleotides predicted by VipR showed that these counts were almost the same as the read counts supporting the other two nucleotides, which were likely to be produced by sequencing errors. Regarding other types of variation, VipR and SNVer were not designed to call variants beyond SNPs. Finally, the output VCF format provided by both tools was largely outdated, which made us feel reluctant of the sustainability of these tools over time. In this scenario, we considered a good alternative to try all the options that we had available, and compare the variants obtained using the different pipelines. As expected, the commonly used tools for variants discovery reported between 4 and 13 times less variants than VipR. A comparison between them was consistent with a previous benchmark that we performed using GBS data, in which NGSEP identifies more SNPs than the other tools [52]. In this case, a possible reason for this difference is that Samtools, GATK and Freebayes were designed to analyze WGS data of human samples. Hence, the models implemented in these tools include filters of balance between read alignment strands, which are not adequate for analysis of reads taken from region-specific PCR products. It is worth to clarify that in the absence of a gold-standard dataset, the comparison presented in this manuscript is not a formal benchmark between methods but a survey of the available alternatives performed from a user perspective. We believe that the results presented in this survey would be helpful for other researchers performing pooled resequencing experiments and also that improved methods for variants discovery in pooled samples could be developed to take full advantage of the data generated by similar experiments.

The final outcome of the comparison between pipelines for variants discovery and the filtering process, including the filtering of variants in which the minor allele could not be clearly separated from sequencing errors, is a dataset of 2614 SNPs within the targeted genes (Supplementary Table 4). Despite of the filtering procedure, close to 80% of these variants are still SNPs with low MAF identified only by VipR. Although we could follow a more conservative approach and report only SNPs called by a certain type of intersection between the tools, this would remove most of the rare mutations that are actually interesting for follow up genotyping experiments. For this reason, we decided to retain the union of the SNPs identified by the different tools after performing the filters described above. However, each SNP is reported with functional annotations, intersection with SNPs obtained from WGS data, predicted allele frequencies, raw read counts and pipelines that reported each variant. This allows different researchers to use common excel filters to select the most appropriate variants for different follow up experiments.

Given the total length of the targeted region, the SNPs identified in this study amount to a density of one SNP for each 26 base pairs. Although we initially found this number surprisingly high, the latest release of the 3000 rice genomes project [53] includes 32 million SNPs for a 400 Mega base pair genome, which corresponds to a density of one SNP for each 12.5 base pairs. In the rice dataset, the number of variants is also increased by accumulation of rare alleles as the sample size increased. Individual genotyping should provide us with a more accurate measure of genetic variability such as the number of pairwise differences per kbp. The *AHAS* genes seem to have larger variability than the genes related to starch production, even after normalization by the covered portion of coding regions. *GBSSI* is the gene with the lowest variability, probably because it is the main enzyme that catalyzes the reaction to produce amylose. Conversely *AHAS4* shows the largest number of variants and also contains three frameshift indels that potentially produce silencing of this paralog. Other interesting variants are the non-sense mutations identified in the single copy *GWD* gene. If these mutations have a silencing effect, plants carrying these SNPs could accumulate abnormally high levels of starch as shown in previous studies [20].

The SNPs identified in this study can be prioritized based on read evidence and predictions of functional consequences, and then they can be tested in a direct genotyping platform. We are currently exploring different alternatives to perform individual genotyping, not only for validation but also to identify varieties with rare alleles that could exhibit interesting characteristics for the traits of interest that then could be selected as new sources of genetic variability for the cassava breeding program. The publication of the SNPs identified in this experiment is helpful to encourage other groups to perform individual genotyping of these SNPs in their own germplasm collections, accelerating the discovery of varieties with improved phenotypes. Moreover, the genetic variation that we could identify in the CIAT collection, within genes that *a*-*priori* could be thought as completely conserved, is also encouraging to try alternative cost-efficient techniques such as multi-dimensional pooled EcoTILLING [54] in future experiments. Although EcoTILLING is in principle a more expensive technique because it requires the design of a tridimensional pooling strategy in which each sample is included in three different pools, it allows direct identification of samples carrying rare alleles. Based on the results of this experiment, we believe that improved methods for targeted resequencing, such as those used in this study, will provide cost-effective valuable information to accelerate breeding cycles through the use of molecular techniques.

## 4 Methods

### 4.1 DNA Extraction

DNA was extracted from a total of 1728 accessions from the germplasm collection at CIAT. The DNA was isolated by using 1 g of cassava leaf tissue grounded with liquid nitrogen in 15 mL tubes using the CTAB method. Thereafter, 3 mL of the prewarmed extraction buffer was added (100 mM tris HCl (pH 8), 20 mM EDTA (pH 8), 2 M NaCl, 2% CTAB (w/v), 2% PVP) to each sample and they were mixed. The samples were incubated at 65 °C for 1 h with frequent swirling. An equal volume of phenol: chloroform: isoamyl alcohol (25:24:1) was added to each sample and mixed gently for 30 min. The samples were centrifuged at 3000 rpm for 30 min at room temperature. Approximately 2 mL of the supernatant was transferred to a new tube. The supernatant was precipitated with 1/1 volume of isopropanol and was incubated for 30 min at 4 °C. The precipitated nucleic acids were collected and washed twice with 70% ethanol. The obtained nucleic acid pellet was air-dried until the ethanol was evaporated and dissolved in 200 uL of TE buffer (10 mM tris-HCl pH 8, 1 mM EDTA pH 8). The nucleic acid dissolved in TE buffer was treated with ribonuclease A (RNase A, 10 mg/mL) and incubated at 37 °C for 30 min. The quality of extracted DNA was stained with SYBR safe (Invitrogen) and visualized by agarose gel electrophoresis (1%). The purity of the DNA was estimated by spectrophotometry, which estimates A260/280 and A260/230 ratio. After this, the dried samples were packed to be shipped to the Plant Breeding and Genetics Laboratory in Austria.

### 4.2 Determination of DNA Quality and Quantity, and Sample Pooling

Once the DNA samples arrived to the Plant Breeding and Genetics Laboratory in Austria for processing and sequencing, were centrifuged and then hydrated by the addition of 100 uL (water). Samples were incubated at room temperature for 10 min followed by a short vortex and an additional 5 min incubation to ensure that DNA was completely in solution. Samples were stored at 4 °C for a minimum of 24 h prior to additional processing.

To ensure even sequencing coverage of all DNA samples in a pool, methods were evaluated to normalize DNA concentrations. Experiments employing paramagnetic bead-based purification systems (*e*.*g*. MagQuantTM) yielded inconsistent concentrations, possibly due to variations of input DNA (data not shown). Therefore a system using 96 well gels and image based quantification was employed [55]. Briefly, 12.5 μL of DNA from each tube was transferred to a well in a 96 well plate to facilitate liquid handling. 5 μL of DNA was loaded onto 96 well E-gels® 2%. Five microliters lambda DNA standards diluted to specific concentrations (3, 4.5, 6.8, 10.1, 15.2, 22.8, 34.2, 51.3 ng/μL) in the last column of the gel. Samples were electrophoresed, the gel photographed and concentrations determined with the aid of the image analysis program ImageJ. Samples' concentrations were adjusted, samples pooled together and the final concentration of each of 8 pools was adjusted to 3.57 ng/μL for PCR.

### 4.3 Primer Design and PCR Performance

A total of 121 primer pairs were designed for the exonic regions of genes related to herbicide tolerance (*AHAS1*, *AHAS2*, *AHAS3*, *AHAS4*, *GS-C1* and *GS-C3*), and starch biosynthesis, (*GWD*, *GBSSI*, *SS-H2*, *SSI*, *SSII*, *SSIII*, *SSIV* and *SBE*). Primer3 [56] was used to design primers with a length between 25 and 30 bp, with a Tm between 65 °C and 72 °C, with an optimal of 70 °C, to amplify fragments between 550 and 650 bp. The TaKaRa Ex Taq® polymerase was used to perform the PCR using 17.85 ng of pooled DNA according to manufacturer's recommendations. Amplification was performed as follows: The initial denaturing cycle was 2 min at 95 °C, followed by 8 cycles of denaturing at 94 °C for 20 s, annealing at 65 °C for 30 s and extension at 72 °C for 1 min. The last cycle extension was held for an extra 5 min, followed by holding at 8 °C. The concentration of PCR products was determined using 96 well E-gels® 1%. PCR products produced from the same DNA were pooled together such that 8 samples of pooled PCR products deriving from the 8 DNA pools created.

### 4.4 Sequencing

Illumina library preparation was performed using the TruSeq® Nano DNA Library Prep (version 15041110 Rev. D) with minor modification. Briefly, the first normalization and fragmentation steps were not performed and library preparation began with the first bead-based cleanup step. All other steps were followed according to the protocol. Dual indexes were used. Quantification was performed using Qubit fluorometry. Libraries were normalized to 4 nM and pooled together. The concentration of this pool was further checked, adjusted, and the pool denatured and diluted to 17.5 pM according to the Illumina protocol. Samples were sequenced on an Illumina MiSeq using 2 × 300 Paired End version 3 chemistry. Fastqc [57] was used to perform an initial quality assessment of the raw reads. The reads did not pass the base quality filter after 240 bp in the first read and after 170 bp of the second read. Accordingly, reads were trimmed to these lengths.

### 4.5 Read Alignment

The reference genome *Manihot esculenta* v6.1 was downloaded from the webpage of Phytozome 11 [58], including the corresponding GFF3 file with gene functional annotations. Two different tools were used to align reads to the reference genome: bowtie2-2.2.5 [42] and BWA 0.7.12-r1039 [43]. The alignment using bowtie2-2.2.5 was made according to the documentation, indexing the cassava reference genome first. The program was run with default parameters, except for the maximum number of alignments per read, which was set to 3, the minimum fragment length to 0 and the maximum fragment length to 800. Picard-2.2.4 [59] was used to sort the BAM files. BWA 0.7.12-r1039 was also used to align reads to the reference genome according to the documentation. The program was executed with the default parameters, setting the bandwidth for banded alignment to 600. Samtools 1.3.1 was used to convert the SAM files into BAM files, to sort them and index them. Visualization of read alignments was performed using the Integrative Genomics Viewer (IGV) [60].

### 4.6 SNP Discovery

Seven variant callers were combined with the two read alignment tools to obtain twelve different pipelines. The procedure for each pipeline is briefly described below.

#### 4.6.1 Freebayes

Freebayes v1.0.2-33-gdbb6160 [44] was executed only from BAM files generated by BWA, according to the documentation available in the website. Samtools-1.3.1 was used to merge the VCF file obtained from each pool and create a final VCF file containing the information of the eight samples. This variant caller could not be executed using files obtained with bowtie2.

#### 4.6.2 GATK

To run GATK 3.5-0-g36282e4 [45] a Sequence Dictionary had to be created using picard 2.2.4, as well as indexing the reference genome using samtools-1.3.1. The Haplotype Caller option was run to obtain the SNPs present in each sample, with the default parameters, except for read downsampling, which was set to 0. At the end, eight VCF files were obtained, one per sample, with all the information about the SNPs present in each of them. This was followed by the Merge Variants option available in this program to obtain a final VCF with the SNP information of all the samples. It's important to mention, that GATK is only compatible with files obtained from BWA, so it was not possible to use this variant caller with the alignment information obtained with bowtie2.

#### 4.6.3 NGSEP

The NGSEP-3.0.1 [46] pipeline was used to discover SNPs and indels. This pipeline was executed with default parameters, except for the maximum number of alignments allowed to start at the same reference site, which was set to 0. The options to find repetitive regions, CNV, large indels and inversions were turned off during the variants discovery and the genotyping steps of the pipeline. Because NGSEP is compatible with bowtie2 and BWA, the pipeline was run with the files obtained with these two alignment programs, with the same parameters mentioned above.

#### 4.6.4 Samtools

The variant calling was performed according to the documentation (version 1.3.1) [47]. Mpileup files were generated and the multi allelic variant caller option was used to detect SNPs. At the end of this process, eight VCF files with the SNP information of each sample were obtained, and the program was used to merge them to obtain a final VCF with the information of all the SNPs present. Because Samtools is compatible with alignment files obtained with bowtie2 and BWA, the same pipeline was run using the different alignment files.

#### 4.6.5 SNVer

SNVer-0.5.3 [48] was executed according to the documentation available. To run this variant caller, a file with five columns that contained the sample name information, number of haploids per pool, number of samples, minimum quality and maximum base quality values, respectively had to be created. At the end, a final VCF file with the information of all the samples was obtained. Because SNVer is compatible with bowtie2 and BWA, this pipeline was run with the information obtained with these two alignments tools.

#### 4.6.6 VarScan

To run VarScan v2.3.9 [49], the documentation available was followed. Mpileup files had to be created first using Samtools. With these mpileup files one of the tools available on the VarScan folder was used to detect the SNPs present in each sample, so at the end of this process eight VCF files with the SNP information were obtained. These files were merged using Samtools to obtain a final VCF file. Because VarScan is compatible with bowtie2 and BWA, this pipeline was run with the files obtained with these two alignment tools.

#### 4.6.7 VipR

This program was executed according to the documentation available (version 0.0.16) [50]. First mpileup files had to be created with Samtools, using the parameters recommended for the documentation. These mpileup files had to be converted into a vipR files. Then, an R script was run following the documentation, setting the number of haploids to 536, corresponding to the biggest pool created in the experiment. At the end, a final VCF file with all the SNP information of each sample was obtained. Because VipR is compatible with bowtie2 and BWA, this pipeline was run with the files obtained with these two alignment tools.

### 4.7 Downstream Analysis

At the end 12 VCF files were obtained as a result of the combination of alignment files made with bowtie2 and BWA and the 7 variant callers. With these 12 VCF files the NGSEP pipeline was used to do the genotyping, first merging the variants present in all the VCF files, and then running the genotyping process with default parameters, except for the maximum number of alignments allowed to start at the same reference site, which was set to 0. This was done with the BAM files for each read alignment tool, generating two final VCF files.

The functional annotation was performed using NGSEP and SNPeff [61], having the GFF3 cassava file as a reference. NGSEP was also used to filter this final file, removing the variants embedded in indels first, and then filtering to keep biallelic SNPs with a read depth of 10000 × or more and those which were present in at least two pools. A custom script written in java was used to filter variants in which the read count of the minor allele is less than five times the read count of the average between the read counts of the third and the fourth allele. Custom scripts were also written to calculate statistics related to the coverage of genes and primers.

## Appendix A Supplementary data

Supplementary Table 1 List of cassava accessions sequenced in this study. Supplementary Table 2 List of genes related to starch content and resistance to herbicides chosen for pooled targeted resequenced. Supplementary Table 3 List of primer pairs used to define the regions targeted for pool resequencing. The last eight columns represent the number of fragments reliably assigned to each region within each pool. Supplementary Table 4 Report of curated biallelic SNPs identified in this study. The table includes for each SNP the chromosome location, reference allele, alternative allele, functional annotation, intersection with the panel of SNPs available at the phytozome website, predicted allele frequencies within each pool, raw A,C,G,T counts within each pool, and pipelines in which the SNP was discovered. Supplementary Table 5 List of curated indels identified in this study. The table includes for each SNP the chromosome location, reference allele, alternative allele, functional annotation, predicted allele frequencies within each pool and raw allele counts for the reference and alternative alleles. Image 1

PDF file including the supplementary figures referred in this study. Image 2
