# Supplementary material for: Deep Assessment of Genomic Diversity in Cassava for Herbicide Tolerance and Starch Biosynthesis
Source: Comput Struct Biotechnol J. 2017 Jan 14;15:185–94. doi: 10.1016/j.csbj.2017.01.002 (PMC5295625; doi:10.1016/j.csbj.2017.01.002)

Supplementary tables and figures for

Deep assessment of genomic diversity in cassava for herbicide tolerance and starch biosynthesis

Jorge Duitama[a,d,e], Lina Kafuri[b,c], Daniel Tello[b,c], Ana María Leiva[a], Bernhard Hofinger[b], Sneha Datta[b], Zaida Lentini[c], Ericson Aranzales[a], Bradley Till[b,f], Hernán Ceballos[a,f]

[a] Agrobiodiversity Research Area. International Center for Tropical Agriculture (CIAT), Cali, Colombia

[b] Plant Breeding and Genetics Laboratory, Joint FAO/IAEA Division, International Atomic Energy Agency, Seibersdorf, Austria

[c] Department of Biological Sciences, School of Natural Sciences, Universidad Icesi, Cali, Colombia

[d] Systems and Computing Engineering Department, Universidad de los Andes, Bogotá, Colombia

[e] Corresponding author. Address: Cra 1 Este No 19A - 40, Bogotá, Colombia. Tel: +5713394949. Email: ja.duitama@uniandes.edu.co

[f] These authors contributed equally to this work and should be considered joint last authors.

Supplementary table 1. List of cassava accessions sequenced in this study

Supplementary table 2. List of genes related to starch content and resistance to herbicides chosen for pooled targeted resequenced

Supplementary table 3. List of primer pairs used to define the regions targeted for pool resequencing. The last eight columns represent the number of fragments reliably assigned to each region within each pool.

Supplementary table 4. Report of curated biallelic SNPs identified in this study. The table includes for each SNP the chromosome location, reference allele, alternative allele, functional annotation, intersection with the panel of SNPs available at the phytozome web site, predicted allele frequencies within each pool, raw A,C,G,T counts within each pool, and pipelines in which the SNP was discovered.

Supplementary table 5. List of curated indels identified in this study. The table includes for each SNP the chromosome location, reference allele, alternative allele, functional annotation, predicted allele frequencies within each pool and raw allele counts for the reference and alternative alleles.

Supplementary Figure 1. 2% agarose E-gel® image for DNA quality evaluation of the samples sequenced in this study

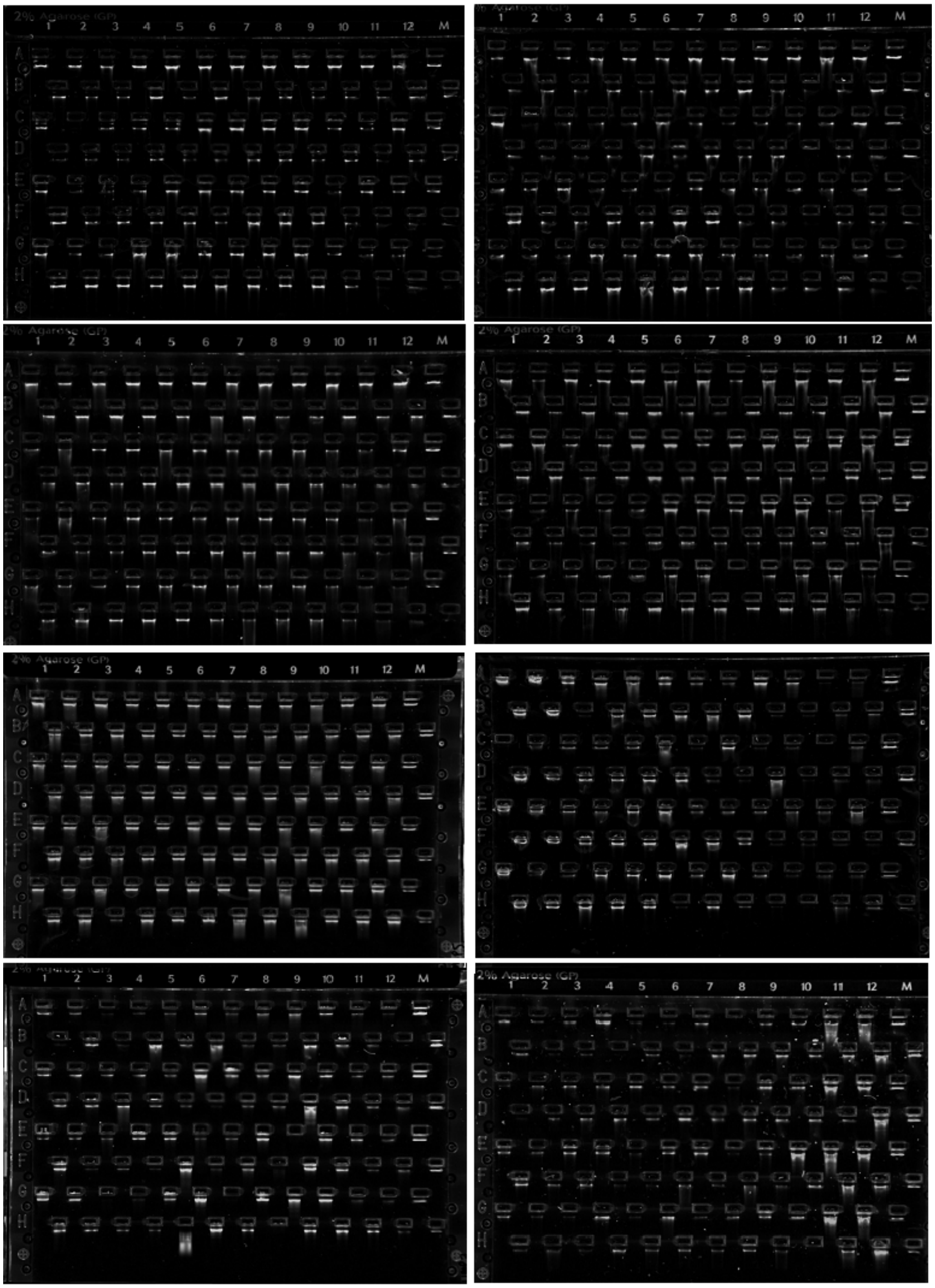

Supplementary Figure 1 (continued)

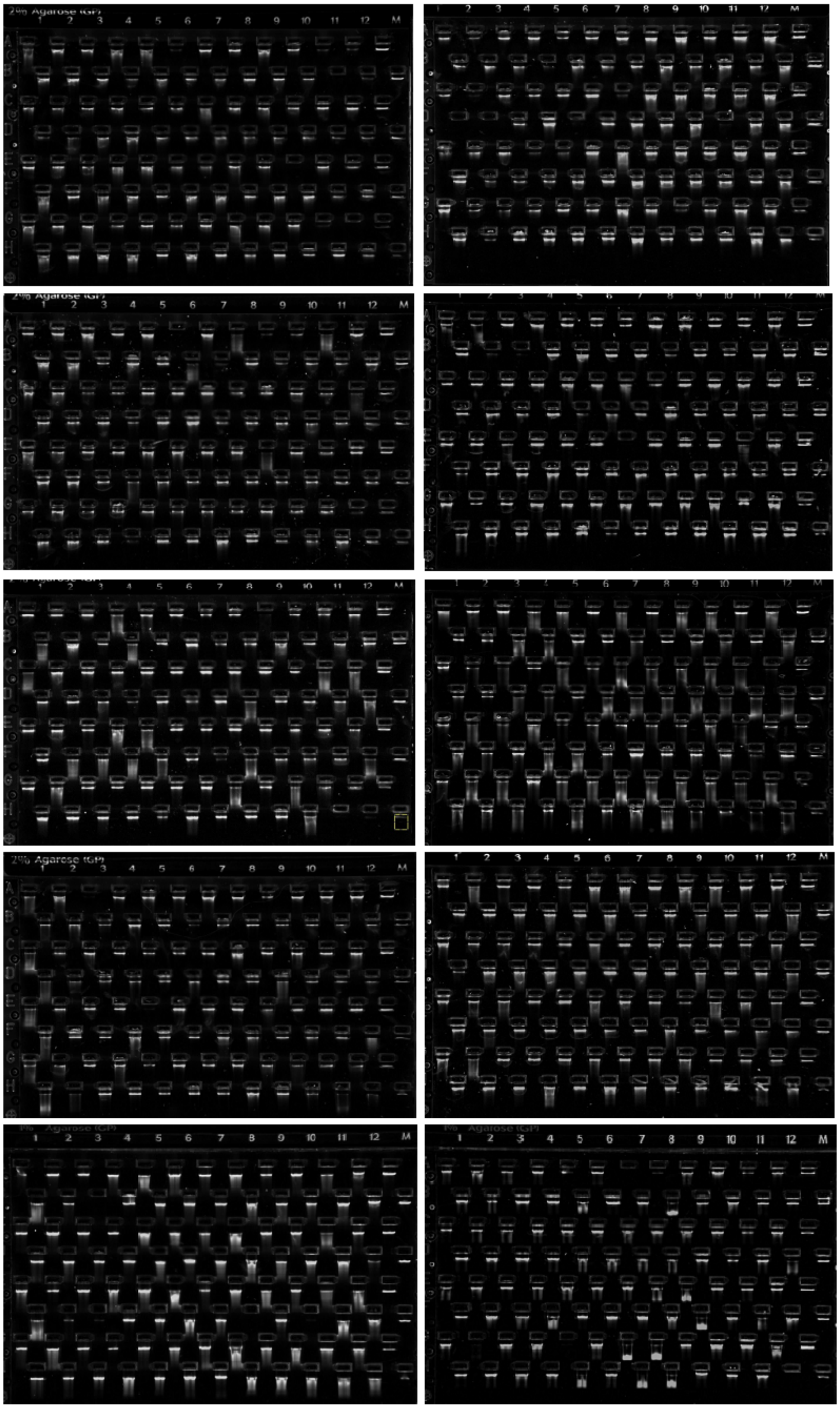

Supplementary figure 2. 1% agarose E-gel® image for PCR amplification evaluation for the 121 primer pairs designed in this study

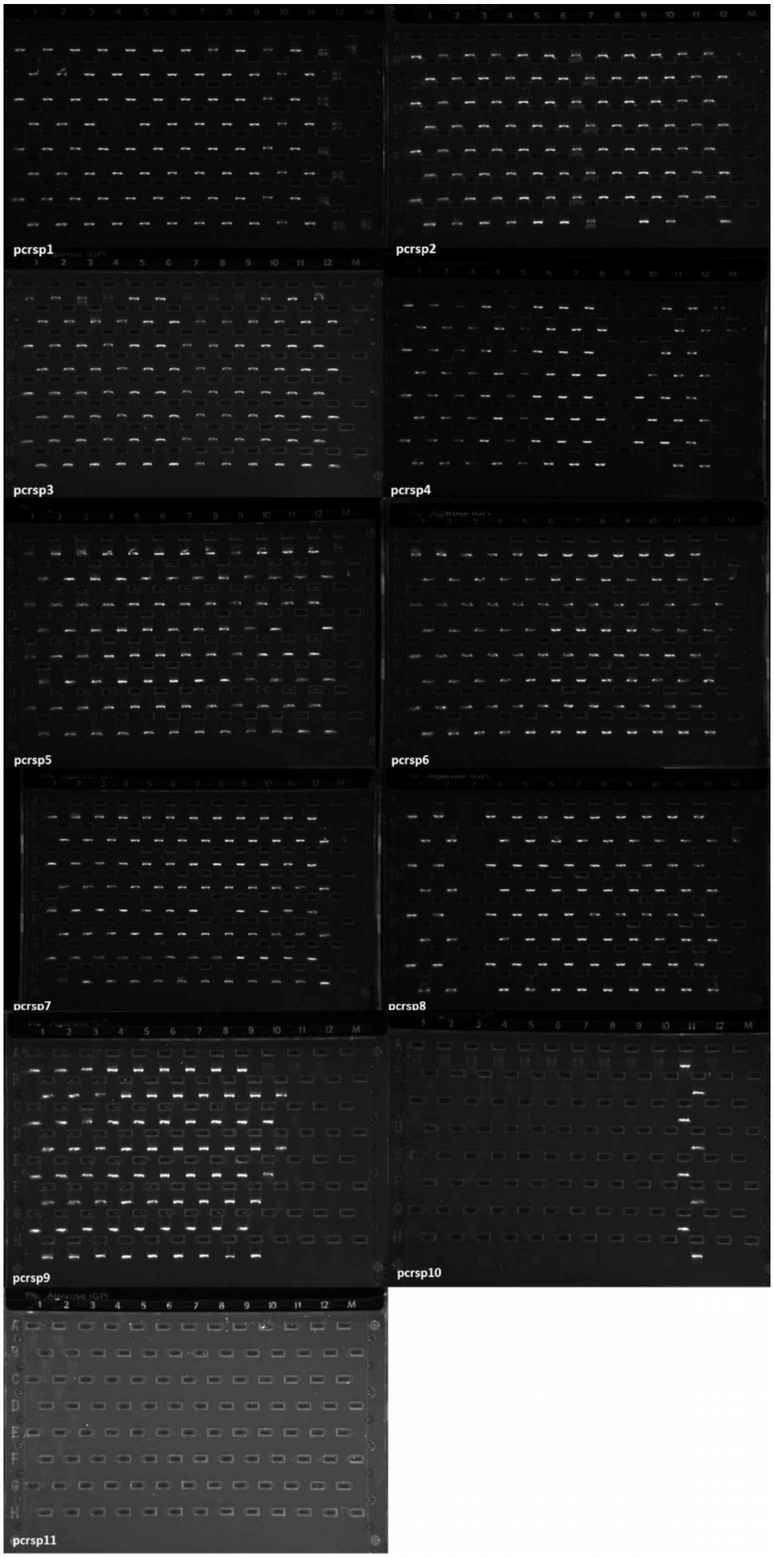

Supplementary Figure 3. Distribution of read depth within each of the 8 sequenced pools for the raw dataset of variants identified in this study. Each datapoint represents the count of reads covering one variant site within one pool. Calls are discriminated by location within or outside targeted regions.

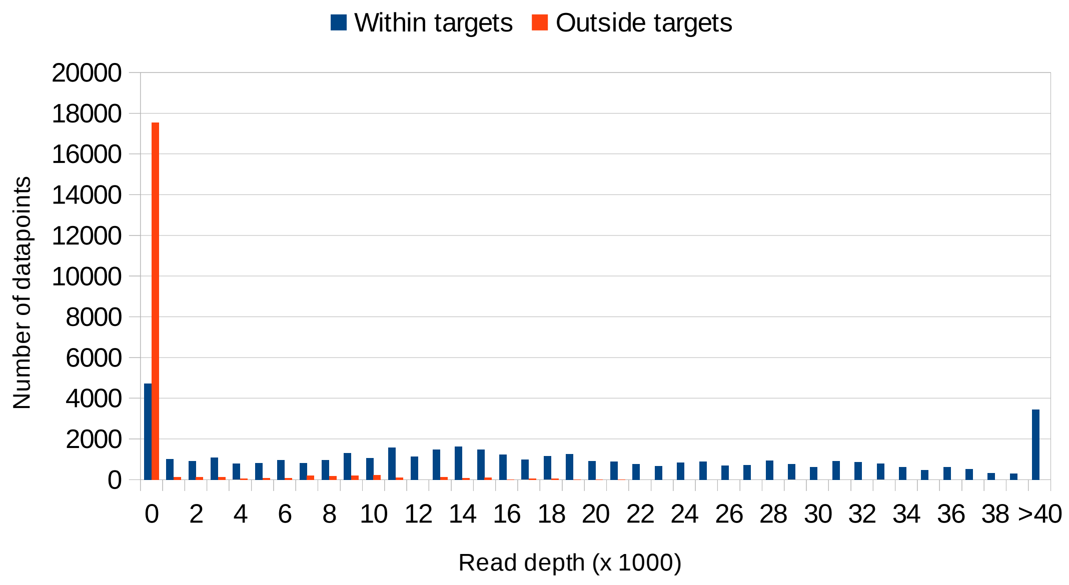

Supplementary figure 4. Percentage of the exonic regions of each gene that could be covered by at least 10,000 reads

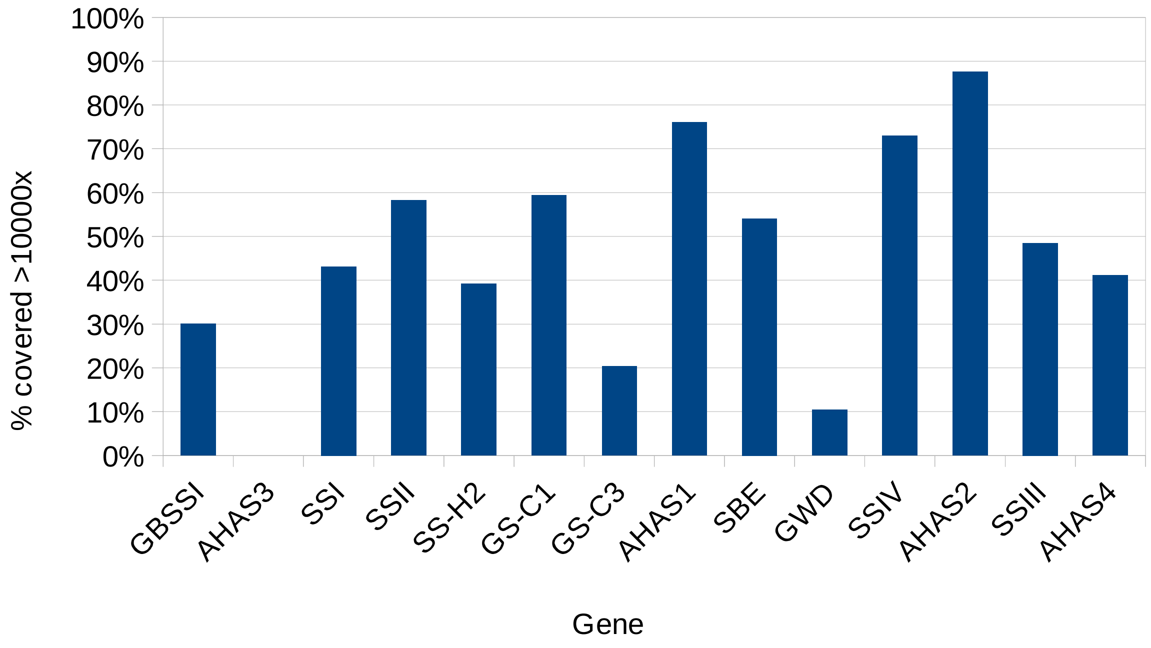

Supplement: Supplementary file 2 — PDF file including the supplementary figures referred in this study. [file mmc2.pdf]
